# Improving Outcomes Best Practices for Pregabalin Withdrawal Management in Hospitals

**DOI:** 10.1192/bjo.2026.11952

**Published:** 2026-06-30

**Authors:** Adam Flynn, Billy Gregg

**Affiliations:** 1 Northern Ireland Medical & Dental Training Agency, Belfast, United Kingdom; 2 Northern Health & Social Care Trust, Antrim, United Kingdom

## Abstract

**Aims:**

Pregabalin dependence and overuse are a major problem, with individuals often taking doses well above the licensed daily maximum of 600mg. Users frequently combine it with other substances, raising the risk of respiratory depression. The lack of guidance on managing withdrawal from high doses poses a significant challenge in hospital settings. Failure to treat acute withdrawal can lead to severe side effects, including seizures, and may result in patients prematurely discharging themselves.

This abstract reviews the limited guidance on assessing and treating pregabalin withdrawal in hospital settings, proposing a structured, evidence-based approach. Our hypothesis is that this approach can safely mitigate withdrawal symptoms and improve patient outcomes.

**Methods:**

The assessment should include evaluating dependence severity and screening for co-existing substance use or mental health disorders. Clinicians should also review clinical notes for previous admissions and, with patient consent, speak with family members for insight into the patient’s drug use. Contacting the local addiction service is important, especially if the patient is on opioid substitute treatment. Point of Care Testing for pregabalin and other drugs can be used, with a positive result supporting a decision to prescribe.

**Results:**

When prescribing pregabalin, the reported dose and objective signs of withdrawal are key. For a reported daily dose of 300-600mg, prescribe 100mg three times daily. For doses over 600mg, an initial dose of 150mg three times daily is recommended, increasing to a maximum of 200mg three times daily if withdrawal persists. Due to common polysubstance use, prescribing diazepam 5mg four times daily is also often required.

**Conclusion:**

Managing high-dose pregabalin withdrawal requires a cautious approach. Patients must be observed for sedation or respiratory depression. Referral to a Mental Health Liaison Service is crucial for advice on tapering and continuity of care. This approach facilitates safer withdrawal and improved outcomes.

